# Auto-Assembling Detoxified Staphylococcus aureus Alpha-Hemolysin Mimicking the Wild-Type Cytolytic Toxin

**DOI:** 10.1128/CVI.00091-16

**Published:** 2016-06-06

**Authors:** Luigi Fiaschi, Benedetta Di Palo, Maria Scarselli, Clarissa Pozzi, Kelly Tomaszewski, Bruno Galletti, Vincenzo Nardi-Dei, Letizia Arcidiacono, Ravi P. N. Mishra, Elena Mori, Michele Pallaoro, Fabiana Falugi, Antonina Torre, Maria Rita Fontana, Marco Soriani, Juliane Bubeck Wardenburg, Guido Grandi, Rino Rappuoli, Ilaria Ferlenghi, Fabio Bagnoli

**Affiliations:** a GSK Vaccines, Research Center, Siena, Italy; b Departments of Pediatrics and Microbiology, University of Chicago, Chicago, Illinois, USA; c Novartis Vaccines and Diagnostics, now GSK Vaccines, Siena, Italy; Food and Drug Administration

## Abstract

Staphylococcus aureus alpha-hemolysin (Hla) assembles into heptameric pores on the host cell membrane, causing lysis, apoptosis, and junction disruption. Herein, we present the design of a newly engineered S. aureus alpha-toxin, HlaPSGS, which lacks the predicted membrane-spanning stem domain. This protein is able to form heptamers in aqueous solution in the absence of lipophilic substrata, and its structure, obtained by transmission electron microscopy and single-particle reconstruction analysis, resembles the cap of the wild-type cytolytic Hla pore. HlaPSGS was found to be impaired in binding to host cells and to its receptor ADAM10 and to lack hemolytic and cytotoxic activity. Immunological studies using human sera as well as sera from mice convalescent from S. aureus infection suggested that the heptameric conformation of HlaPSGS mimics epitopes exposed by the cytolytic Hla pore during infection. Finally, immunization with this newly engineered Hla generated high protective immunity against staphylococcal infection in mice. Overall, this study provides unprecedented data on the natural immune response against Hla and suggests that the heptameric HlaPSGS is a highly valuable vaccine candidate against S. aureus.

## INTRODUCTION

Staphylococcus aureus is a major human pathogen, and current antibiotics are not efficacious against emerging multidrug-resistant strains. Therefore, there is an urgent need to develop vaccines to target this pathogen. However, S. aureus vaccine development is hindered by the lack of known correlates of protection. Alpha-toxin, also known as alpha-hemolysin (Hla), is one of the major virulence factors of S. aureus. The toxin is secreted as a water-soluble monomer and can bind to erythrocytes, platelets, monocytes, lymphocytes, and endothelial as well as epithelial cells (1, 2). Monomers assemble into a membrane-perforating homoheptamer upon binding to its eukaryotic proteinaceous cellular receptor, ADAM10 (1, 3–5). Hla-based vaccines have been previously described (2, 6, 7). Nonetheless, all published studies on Hla have so far used monomeric mutants of Hla without knowing if they were able to expose epitopes resembling the heptameric conformation of the active toxin, which is assembled during infection on host cells.

Herein, we present a study aimed at investigating the structural, functional, and immunogenic characteristics of monomeric versus heptameric Hla. We engineered the toxin by removing the membrane-spanning stem domain. This protein, HlaPSGS, is able to form heptamers in aqueous solution in the absence of a host cell membrane and resembles the cap of the wild-type cytolytic Hla pore. Therefore, we can use this mutant to understand if the immune response of the host during infection is more prominent to the monomeric or the heptameric form of Hla. Immunological studies using human sera as well as sera from mice convalescent from S. aureus infection suggested that the heptameric conformation of HlaPSGS mimics epitopes exposed by the cytolytic Hla pore during infection.

Furthermore, we evaluated the molecule as a vaccine antigen against S. aureus. HlaPSGS was found to lack hemolytic and cytotoxic activity and also to be impaired in binding to host cells. In addition, immunization with HlaPSGS generated high protective immunity against staphylococcal infection in mice.

Overall, these data suggest that host-pathogen interactions that occur during infection with S. aureus generate an immune response, in humans and mice, directed primarily against conformational epitopes that are present only in the heptameric Hla toxin. Therefore, this study provides important observations for understanding the immunology and pathogenesis of S. aureus infections as well as for developing innovative vaccines.

## MATERIALS AND METHODS

### Bacterial strains, media, and growth conditions.

For mouse infection studies, overnight cultures of the S. aureus LAC (USA300) strain were diluted 1:100 into fresh tryptic soy broth (TSB) and were grown at 37°C with shaking until reaching mid-log phase. Bacteria were centrifuged, washed with phosphate-buffered saline (PBS), and suspended in a volume of PBS to yield the appropriate cell concentration, which varied according to the model used. The inoculum was verified experimentally by plating on tryptic soy agar (TSA) and colony enumeration.

For expression of Hla constructs, Escherichia coli was grown in Luria Bertani broth that contained 30 μg/ml kanamycin until an optical density (OD) of 0.4 and was then induced with 1 mM isopropyl-β-d-thiogalactopyranoside (IPTG) and grown for 3 more hours at 25°C.

### Cloning of Hla constructs.

*hla* was amplified by PCR from the S. aureus NCTC8325 strain using oligonucleotides HlaF/HlaR. HlaPSGS was amplified by splicing by overlapping extension PCR (SOE PCR) from the S. aureus NCTC8325 strain using oligonucleotide pairs HlaF/PSGSR and PSGSF/HlaR and then the pair HlaF/HlaR. HlaH35L was amplified by SOE PCR from the S. aureus NCTC8325 strain using oligonucleotide couples HlaF/H35LR and H35LF/HlaR and then the couple HlaF/HlaR.

The HlaPSGS and HlaH35L constructs were cloned as N-terminal 6His-tagged constructs into the pET-15b+ vector using the polymerase incomplete primer extension (PIPE) technique (8). *hla* and *hlaPSGS* genes were also amplified by PCR using oligonucleotides HlanatF/HlanatR and were cloned as tagless constructs into the NdeI/XhoI sites of the pET-24b+ vector. All of the constructs were transformed in E. coli BL21(DE3). For primers used in this study, see Table S1 in the supplemental material.

Hla epitopes were generated as previously described (9).

### Purification of Hla recombinant proteins.

Escherichia coli cells expressing HlaPSGS, HlaWt, or HlaH35L (Hla recombinant proteins) were collected by centrifugation, suspended in a 50 mM potassium phosphate buffer (pH 7.0), and disrupted by ultrasonic oscillation at 4°C for 20 min with a Seiko Instruments ultrasonic disintegrator model 7500. Cell debris was removed by centrifugation. The supernatant solution was suspended in 50 ml of 30 mM Tris (pH 9.5) and was applied to a Q Sepharose HP ion exchange column (GE Healthcare) that was previously equilibrated in the same cell suspension buffer. Flowthrough fractions containing Hla recombinant proteins were collected and pooled. The pooled protein solution (20 ml) was diluted 1:3 (i.e., to a final volume of 60 ml) with 10 mM Na phosphate (pH 6.8) and was applied to a 20-μm hydroxyapatite column (Bio-Rad) that was previously equilibrated with 10 mM Na phosphate (pH 6.8), and a 10 mM Na phosphate (pH 6.8) to 1 M Na phosphate (pH 6.8) gradient was applied. Hla recombinant proteins came out in the flowthrough fractions, which were pooled, while most impurities were eluted during the gradient and discarded. A 500 mM Na phosphate solution was added to the pooled protein solution in order to bring the phosphate concentration to 50 mM, and pH was corrected to 6.3. This protein solution was loaded on an SP Sepharose HP ion exchange column (GE Healthcare) that was previously equilibrated in 50 mM Na phosphate (pH 6.3). Flowthrough fractions that contained Hla recombinant proteins were pooled. Fractions were concentrated by ultrafiltration and were loaded on a Superdex 200 26/60 (GE Healthcare) size-exclusion chromatography column equilibrated in phosphate-buffered saline. Gel filtration fractions were selected by SDS-PAGE and size-exclusion chromatography with multiangle laser light scattering (SEC-MALLS) and were pooled.

### Protein purity analysis.

Purity and apparent molecular weight (MW) of HlaWt and HlaPSGS proteins were determined by an analytical size-exclusion high-pressure liquid chromatograph (HPLC) TSK G3000SWxl (7.8 by 300 mm; Tosoh Bioscience Corporation) with isocratic elution in PBS at a flow rate of 0.5 ml/min. UV absorbance was monitored at 280 nm. For molecular mass determination, a gel filtration standard (670, 158, 44, 17, and 1.35 kDa; Bio-Rad) was applied.

### Erythrocyte ghost membrane preparation.

Erythrocyte ghost membranes were prepared as previously described (10).

### Molecular modeling.

Structural models of monomeric HlaWt and HlaPSGS were obtained by computer modeling using the crystal structure of the monomeric Hla as a template. Heptameric HlaPSGS was built onto the crystal structure of heptameric HlaWt (4). Swiss-PdbViewer (11) version 3.5b was used to generate all of the structural models by torsion angle manipulation and energy minimization. Default parameters were used to satisfy the spatial restraints. The stereochemical validity of the final models was confirmed using ProCheck (12).

### Electron microscopy.

A 5-μl aliquot of purified protein with a concentration of 0.10 μg/μl was incubated for 90 min at 37°C with 25 μl purified rabbit erythrocyte ghost membrane, and then a 5-μl aliquot of the mixture was applied to 300-square mesh Formvar nickel grids coated with a thin carbon film and left to stand for 5 min. Excess solution was blotted with Whatman filter paper (catalog no. 1001150). The grids were first washed by streaming several drops of PBS over the grids and were then negatively stained with two drops of 1% buffered ammonium molybdate (AMb), pH 7. The last drop was left on the grids for 40 s. Finally, the grids were washed with several drops of double-distilled water (ddH_2_O), the excess liquid was soaked off by Whatman filter paper, and the grids were air dried. The grids were observed using a transmission electron microscope (TEM) FEI Tecnai G2 Spirit operating at 80 kV and equipped with a charge-coupled-device (CCD) camera Olympus SIS Morada (2,000 × 2,000 pixels).

### Image processing.

Single particles were semiautomatically picked from digitized images using the Boxer tool from the EMAN software package (13). Images were first cut into individual boxes of 128 by 128 pixels, band-pass filtered with a Gaussian edge at 17 to 200 Å to remove the background, and then normalized using Imagic 5 version 2010 (14). The reconstruction was performed by using ∼10,000 particles that were taken from 40 micrographs of HlaPSGS in the absence of ghost membrane substrate at 80 keV at a nominal magnification of ×220,000. Approximately 5,000 particles were discarded from the initial set of particles. The elimination process was performed manually during the reconstruction by comparing each particle to others in approximately the same orientation and keeping only the most self-consistent data. Boxed particles were rotated, translated, and centered and were then classified by multivariate statistical analysis (MSA) to sort images into class averages with similar features. Euler angles were assigned to class averages that were used to reconstruct an initial three-dimensional (3D) map using Imagic 5 version 2010. The initial 3D map was than refined by adding class averages of the side views as reprojections from the initial 3D map. Iterative cycles of angular reconstitution and 3D reconstruction were applied until the Euler angle values were stable. The final 3D map was refined at a 30-Å resolution (Fourier shell correlation [FSC], 0.5) according to EMAN or at a 28-Å resolution (FSC, ½ bit) according to Imagic 5 version 2010. Surface representations rendered in 3D were visualized in UCSF Chimera (15).

### Transepithelial electric resistance measurements.

To measure the maintenance of the integrity of epithelial monolayer permeability during incubation with HlaWt and HlaPSGS, we used the xCELLigence system (Roche). To avoid possible interference of the His tag with the cell to cell junction dissociation activity of Hla, tagless versions of HlaWt and HlaPSGS were used. The instrument monitors cellular events in real time and measures electrical impedance across interdigitated microelectrodes integrated on the bottom of tissue culture E-plates. The impedance measurement provides quantitative information about the biological status of the cells, including cell number, viability, and morphology. Briefly, A549 cells were plated on E-plates and grown to full confluence as monitored by reaching transepithelial resistance (TER) stability for 1 day. Cells were then incubated with 100 μg/ml Hla and 100 μg/ml HlaPSGS. Streptolysin O (SLO), a well-known pore-forming toxin that is produced by Streptococcus pyogenes, was used as a positive control at a concentration of 20 μg/ml, while PBS was used as a negative control. These concentrations were selected during experimental setup on the basis of the ability of the two wild-type toxins to reach the plateau in terms of TER reduction. Measurements were taken in triplicate (mean ± standard deviation [SD]) and reported as an arbitrary cell index.

### Binding assay.

Binding of recombinant purified HlaWt and HlaPSGS to A549 cells (human lung carcinoma epithelial cells) was revealed with mouse polyclonal antibodies against the proteins and with R-phycoerythrin-conjugated goat anti-mouse IgG as secondary antibody. To avoid possible interference of the His tag with the cell binding ability of Hla, tagless versions of HlaWt and HlaPSGS were used. As a negative control, cells were incubated with primary polyclonal antibodies that were detected by fluorescence-labeled secondary antibodies or by fluorescence-labeled secondary antibodies alone. In order to quantify binding, fluorescent intensity was measured by a fluorescence-activated cell sorter (FACS), and data were expressed as mean fluorescence intensity (subtracted from the fluorescence measured in the negative controls). The analysis was performed by CellQuest software and histograms relative to the intensity of fluorescence based on a number of ≥10,000 events. Binding experiments were performed at 4°C for 1 h.

### Coimmunoprecipitation studies.

Human alveolar epithelial cells (A549; 1.5 × 10^7^ per condition) were resuspended in 1 ml of PBS and then treated with 5 μg of recombinant toxin for 20 min at room temperature. Following incubation, cells were pelleted, supernatants were removed, and pellets were solubilized in 1 ml of radioimmunoprecipitation assay (RIPA) lysis buffer (50 mM Tris [pH 7.4], 150 mM NaCl, 0.1% SDS, 1% deoxycholate, 1% Triton X-100, complete EDTA-free protease inhibitor [Roche]) for 10 min on ice. Lysates were clarified by centrifugation (13,000 rpm for 10 min at 4°C), and immunoprecipitations were performed by incubating the lysates with 5 μl of anti-Hla rabbit polyclonal serum (6) for 2 h on ice followed by precipitation of immune complexes with protein G agarose (Pierce) at 4°C for 1 h. Samples were washed three times in 1 ml of RIPA buffer and were then resuspended in nonreducing Laemmli sample buffer and separated by SDS-PAGE. For Western blotting analysis of ADAM10, the blot was blocked overnight at 4°C (5% milk in Tris-buffered saline containing 0.1% Tween 20) and probed with 1 μg/ml of goat anti-ADAM (R&D Systems) and mouse anti-ADAM10 monoclonal antibody (R&D Systems) for 1 h at room temperature. Alexa Fluor 680-conjugated anti-goat and anti-mouse secondary antibodies were utilized to facilitate protein detection in the Odyssey LI-COR infrared imaging system. For Hla immunoblotting, blots were reblocked overnight (5% milk in Tris-buffered saline containing 0.1% Tween 20) and were probed with anti-Hla polyclonal rabbit serum followed by goat anti-rabbit Alexa Fluor 680 prior to imaging.

### Hemolysis assay.

Erythrocytes derived from defibrinated rabbit blood were suspended in 5 ml PBS and placed on an orbital shaker at room temperature until used. Microplates were filled with 150 μl of recombinant antigens in PBS and 50 μl of erythrocytes. The concentration of erythrocytes in rabbit blood is roughly 5 × 10^12^ to 6 × 10^12^ per liter (16). To avoid possible interference of the His tag with the hemolytic activity of Hla, tagless versions of HlaWt and HlaPSGS were used. As a negative control, erythrocytes were incubated with 10 mM K_2_H_2_PO_4_ and 150 mM NaCl plus 0.5% bovine serum albumin (BSA). Incubation with water plus 1% Triton X-100, which causes osmotic lysis, was the positive control of the test. Proteins were diluted in 10 mM K_2_H_2_PO_4_ and 150 mM NaCl plus 0.5% BSA. Plates were then incubated at 37°C for 30 min and centrifuged at 1,000 rpm for 5 min at 4°C. The supernatant was removed and analyzed spectrophotometrically by a SpectraMax 340PC384 absorbance microplate reader (Molecular Devices) at 540 nm. In experiments that tested the ability of purified total IgG against HlaPSGS to prevent the lysis of erythrocytes, we incubated active Hla at a concentration of 100 nM with serially diluted purified total IgG against HlaPSGS or adjuvant alone. Total IgG from polyclonal mouse serum raised against HlaPSGS was purified by an IgG purification column and dialyzed against PBS. Before and after overnight dialysis, antibody concentration was determined by Bradford protein assay. Incubation of purified Hla IgG with purified Hla toxin was performed at room temperature for 20 min before adding erythrocytes to the samples. Plates were then incubated at 37°C for 30 min and centrifuged at 1,000 rpm for 5 min at 4°C. Supernatant was then analyzed spectrophotometrically by a SpectraMax 340PC384 absorbance microplate reader (Molecular Devices) at 540 nm.

### ADAM10 binding assay.

The ^35^S-radiolabeled Hla toxin was synthesized by *in vitro* transcription and translation in E. coli S30 extract (Promega) supplemented with T7 RNA polymerase, rifampin, and [^35^S]methionine according to the manufacturer's instructions. Binding of Hla was assessed using A549 cells at 5 × 10^5^ cells/500 μl incubated with 15 μl of serum collected from HlaPSGS-immunized mice or with serum collected from sham-immunized mice (alum) for 20 min at room temperature. Then, 0.3 nM radiolabeled Hla was added to the cells for 5 min at room temperature. Following incubation of the toxin, 500 μl of cold PBS was added to the cells and then pelleted at 15,000 × *g* for 2 min and washed twice in cold PBS. Cell pellets were then resuspended in 250 μl of PBS and added to scintillation fluid for quantification of bound radioactivity (counts per minute) on a Beckman LS 6000 scintillation counter. Significance was determined using Student's *t* test in Prism.

### Immunization protocol and pneumonia model.

HlaPSGS was formulated in aluminum hydroxide adjuvant (alum, 2 mg/ml), and adjuvant alone was used for immunization of control mice. C57BL/6J mice received two intramuscular immunizations with a prime-booster injection 2 weeks apart and were infected 2 weeks after the second immunization. Eighteen micrograms of HlaPSGS was administered to each mouse. For lung infection, mice were inoculated with a 3 × 10^8^ to 4 × 10^8^ CFU per 30 μl suspension of S. aureus strain LAC into the left naris as previously described (17). Animals were placed into the cage in a supine position for recovery and observed. Survival curves were generated using GraphPad Prism 5 software.

### Luminex assay.

Antibody titers present in sera from immunized or infected mice as well as in sera from human volunteers were measured by Luminex technology (Luminex 200TM). HlaPSGS, HlaWt, and glutathione *S*-transferase (GST) peptides were covalently conjugated to the free carboxyl groups of microspheres using an *N*-hydroxysulfosuccinimide-enhanced carbodiimide-mediated conjugation chemistry. Antigen-specific antibodies were revealed by phycoerythrin-labeled secondary antibodies. The assay read-out is a measure of fluorescence intensity at fixed serum dilution.

### Statistical analysis.

At least two independent experiments, run under the same conditions, were performed for all studies. For the pneumonia models, survival curves were generated by the Kaplan-Meier analysis method, and statistical significance was determined using the log-rank (Mantel-Cox) test. For the ADAM10-binding assay, significance was determined using Student's *t* test in GraphPad Prism 5 software.

### Ethics statement.

Mice were monitored twice per day in order to evaluate early signs of pain and distress according to humane endpoints for the experiment. These signs included respiration rate, posture, and loss of weight (more than 20%). Animals showing such conditions were euthanized in accordance with experimental protocols that were reviewed and approved by the Novartis Animal Welfare Body and the Italian Ministry of Health (protocol no. 136/2010-B for mouse studies and no. 201103 for rabbit studies).

Human sera from healthy subjects were purchased from 3H Biomedical (written informed consent was obtained from each subject in compliance with the world medical association declaration of Helsinki).

## RESULTS

### Designing a noncytolytic heptameric Hla.

**FIG 1:**
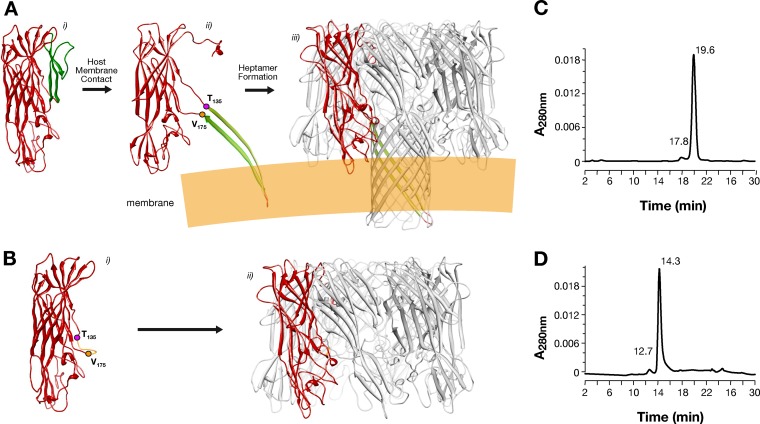
*In silico* design of HlaPSGS and size-exclusion HPLC analysis of Hla proteins. (A) (i) HlaWt protomer based on the crystal structure of the Hla monomer. The stem domain spanning from amino acids T_135_ to V_175_ is colored green. (ii) Following host cell membrane contact, the toxin heptamerizes, and the stem domain of each monomer rearranges simultaneously and inserts into the cell membrane. (iii) The stem domains of the seven protomers form the transmembrane channel. (B) (i) HlaPSGS protomer designed *in silico*. The stem domain was substituted with the four amino acid linker PSGS (colored orange) inserted between residues T_135_ and V_175_. Given the absence of the stem, the protomer can auto-assemble into heptamers independently of host cell membrane contact but without forming a transmembrane channel. (C and D) Size-exclusion HPLC analysis of HlaWt and HlaPSGS. Purity of the proteins as measured by SEC appears to be greater than 95%. (C) The SEC profile of HlaWt presents an elution peak maximum that corresponds to an apparent size of around 35 kDa, indicating that the protein is monomeric in solution. (D) The SEC profile of HlaPSGS presents an elution peak maximum of 14.3 min that corresponds to an apparent size of around 220 kDa, which is compatible with a heptameric state in solution of HlaPSGS.

The *hla* gene encodes the 293-amino-acid protein protomer, which forms complete heptameric beta-barrel pore structures on the cellular membrane (Fig. 1A). According to the structure of the Hla monomer (18), the stem β strands spanning from amino acid Thr^135^ to Val^175^ are oriented upward, which interfers with heptamer formation (Fig. 1A). Upon contact with the host cell membrane, monomers undergo a conformational change, inserting the stem β strands in the cell membrane and forming heptameric pores (Fig. 1A). On the basis of this structural model, the HlaPSGS mutant (Fig. 1B) was designed to replace the 39-amino-acid stem with a short linker composed of the amino acid residues proline, serine, glycine, and serine (PSGS). We predicted two major consequences associated with this replacement: (i) prevention of transmembrane channel formation and therefore full detoxification of the protein and (ii) spontaneous oligomerization in solution even in the absence of the host cell membrane. The newly designed mutant protein and the wild-type Hla (HlaWt) were expressed in the cytoplasm of E. coli and were purified from soluble cell extracts using a four chromatographic step procedure. The protein purity of HlaWt and HlaPSGS was estimated by size-exclusion HPLC (SEC) and was found to be greater than 95% (Fig. 1C and D).

### HlaPSGS auto-assembles in liquid solution to form a heptameric ring-like structure.

Size-exclusion HPLC of HlaWt and HlaPSGS revealed their different assembly statuses in liquid solution. Indeed, the elution peak maximum was found at 19.9 min for HlaWt, which corresponds to an apparent size of about 35 kDa (Fig. 1C), indicating that the protein is monomeric in solution. Conversely, the SEC profile of HlaPSGS presents an elution peak maximum of 14.3 min, which corresponds to an apparent size of around 220 kDa and is compatible with a heptameric state in solution of the molecule (Fig. 1D).

**FIG 2:**
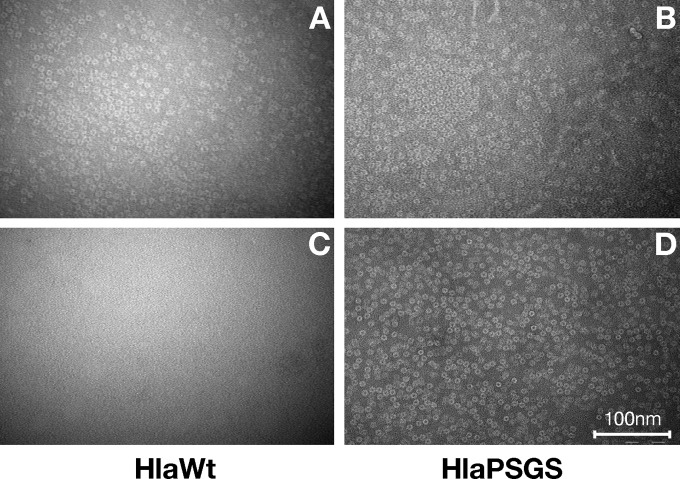
HlaPSGS forms auto-assembling heptameric pores in liquid solution. (A to D) Negative stained micrographs of wild-type Hla and HlaPSGS. Wild-type Hla (A) and HlaPSGS (B) added to rabbit red blood cell membranes show pore structures. Formation of pores with HlaWt and HlaPSGS can be observed after 5 to 10 min of incubation on ghost membranes at 37°C; nonetheless, all micrographs were taken after 90 min of incubation at 37°C. When samples were analyzed in liquid solution, no pore structures were observed with HlaWt (C), while HlaPSGS formed pore structures (D) similar to the ones observed in the presence of rabbit red blood cell membranes (A and B). Scale bar, 100 nm.

**FIG 3:**
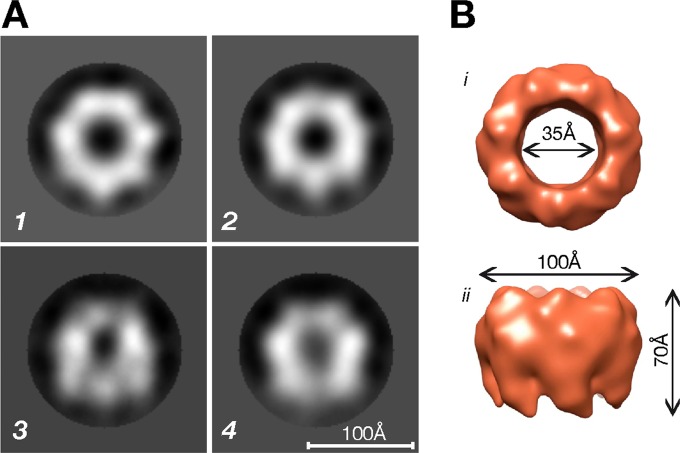
Overall structure of the HlaPSGS heptamer. (A) Typical HlaPSGS class averages clearly showing seven peaks corresponding to the seven identical protomers forming the ring-like HlaPSGS structure. (B) Top (i) and side (ii) surface views of the HlaPSGS electron density map obtained at a 28-Å resolution (FSC, ½ bit). The ring has a seven-spike donut shape, with a clear 7-fold symmetry (C7) and seven thick arms oriented clockwise and protruding from the bottom of the structure. It has an external diameter of 100 Å, a height of 70 Å, and an internal pore diameter of 35 Å. (A) Scale bar, 100 Å.

In agreement with the crystallographic structure (4), transmission electron microscopy (TEM) analysis of HlaWt indicated that in the presence of rabbit erythrocyte ghost membranes the protein forms a heptameric ring-like complex with a diameter of ∼100 Å with smooth circular external edges and an internal pore of ∼30 Å (Fig. 2A). Such structures were never observed when HlaWt was analyzed in the absence of cell membranes (Fig. 2C). In contrast, HlaPSGS formed ring-like structures regardless of the presence of ghost membranes (Fig. 2B and D). In order to trace the overall structure of HlaPSGS and compare it with that of the wild-type Hla, the three-dimensional (3D) structure of the mutant protein was determined by using TEM combined with single-particle reconstruction. About 5,000 particle images contributed to the final reconstruction. Except for the absence of the stem region, the molecular features in the HlaPSGS electron density map were comparable to those observed in the electron density map of the wild-type Hla. The heptameric form of the HlaPSGS ring-like structure was clearly visible even at early stages of the 3D reconstruction (Fig. 3A). The final 3D model, obtained after 2 rounds of iterative refinement (Fig. 3B), was generated at a 28-Å resolution (FSC, ½ bit). The model shows a seven-spike donut shape, with a clear 7-fold symmetry (C7-symmetric pore), a feature common to other toxins known in the literature (19–21). Seven thick arms, oriented clockwise, protrude from the bottom of the structure, which has an external diameter of ∼100 Å, is ∼70 Å in height, and has an internal pore diameter of ∼35 Å (Fig. 3B).

### Wild-type Hla, but not HlaPSGS, binds to ADAM10 and impairs human epithelial cell monolayer integrity.

**FIG 4:**
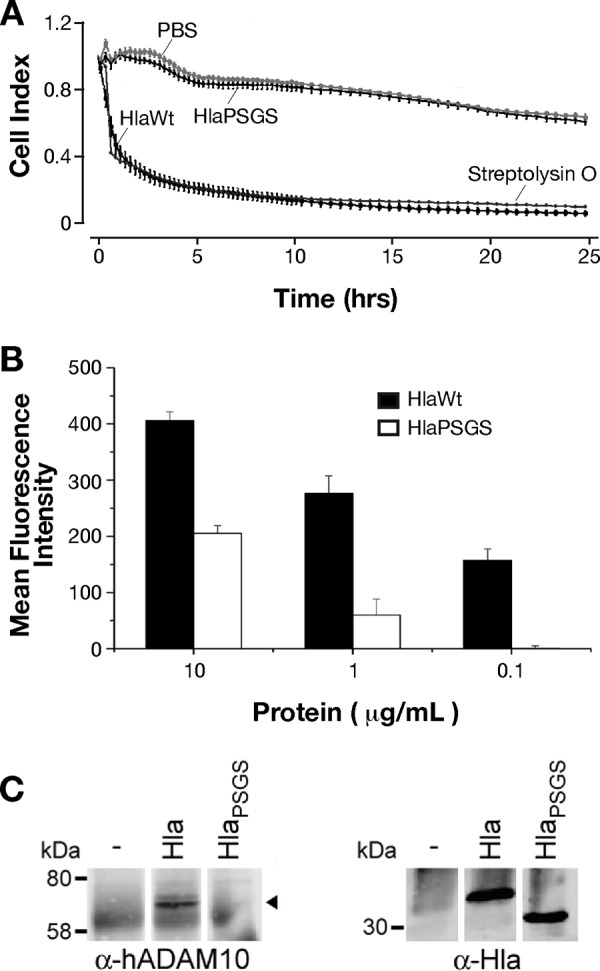
HlaPSGS does not alter the integrity of human alveolar epithelial cell monolayers, and its binding to the cells and the Hla host receptor ADAM10 is impaired. (A) Integrity of the A549 monolayer following treatment with HlaWt and HlaPSGS. Graphs report the transepithelial electric resistance values measured by xCELLigence. HlaWt and HlaPSGS were added at a concentration of 100 μg/ml and SLO at 20 μg/ml. The cell index is an arbitrary unit for electric impedance measurement. Data are expressed as mean ± SD of three independent wells. (B) The ability of HlaWt and HlaPSGS to bind to A549 cells was assessed by an indirect immunofluorescence detection system and was analyzed by FACS. Columns in the plot indicate mean fluorescence intensity ± SD of three independent experiments. (C) HlaPSGS does not interact with ADAM10 on human alveolar epithelial cells. A549 alveolar epithelial cells treated with HlaWt or HlaPSGS were subjected to Hla immunoprecipitation and ADAM10 immunoblotting, demonstrating an interaction of the wild type with ADAM10 (left). In contrast, ADAM10 is not detected in HlaPSGS precipitates (left). Equivalent amounts of HlaWt and the mutant variant were present in each immunoprecipitation (right).

Hla is toxic for epithelial cells and has been recently demonstrated to promote cell junction dissolution by interacting with the cellular protease ADAM10 (3, 22–24). Therefore, we wondered if the absence of the stem domain in HlaPSGS was sufficient to impair these activities. To that end, we measured the electrical impedance across a confluent monolayer of the human alveolar epithelial cell line A549 using the xCELLigence system. Impedance reflects the status of the cell monolayer, including cell confluence, viability, and junction functionality (25). A549 cells were grown to confluence on xCELLigence inserts, and the experiments were started when transepithelial resistance (TER) was found to be constant between two measures taken 24 h apart. Cells were then incubated for 24 h with 100 μg/ml of HlaWt or HlaPSGS. Streptolysin O (SLO), a well-characterized pore-forming toxin produced by Streptococcus pyogenes, was used as a positive control at a concentration of 20 μg/ml, while PBS was used as a negative control. As shown in Fig. 4A, SLO and HlaWt significantly affected epithelial monolayer integrity as indicated by a decrease in TER (quantified by the decrease in arbitrary cell index values), while cells treated with HlaPSGS maintained a constant TER throughout the duration of the experiment.

Furthermore, we observed a striking reduction in the capacity of HlaPSGS in binding to A549 cells compared to that in HlaWt (Fig. 4B). This suggested that removal of the stem domain affects the ability of the toxin to interact with the host cell and ADAM10. To assess this hypothesis, human A549 alveolar epithelial cells were treated with wild-type Hla or HlaPSGS, lysed, and subjected to Hla immunoprecipitation. Precipitated proteins were probed for ADAM10 and the toxin. Wild-type Hla interacted with ADAM10, while the HlaPSGS mutant toxin did not precipitate ADAM10 (Fig. 4C).

### HlaPSGS is not hemolytic, and its antibodies neutralize the activity of the wild-type toxin and binding to human epithelial cells.

**FIG 5:**
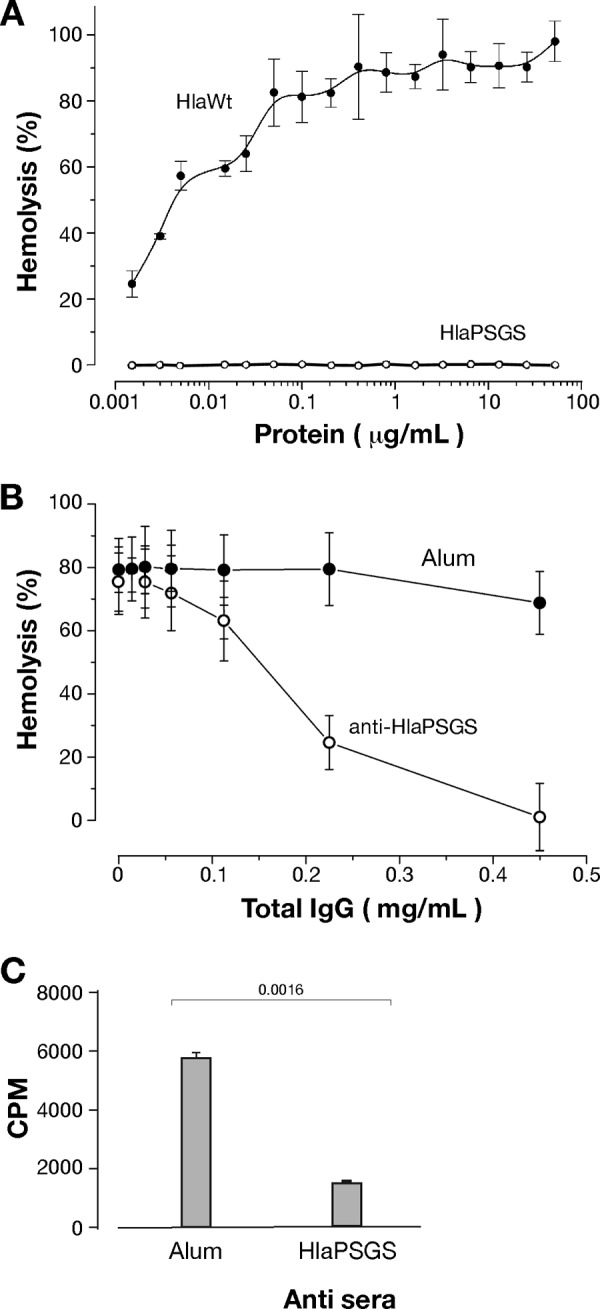
HlaPSGS lacks hemolytic activity, and its antibodies neutralize wild-type Hla-induced hemolysis and the binding of the toxin to A549 cells. (A) Rabbit blood hemolysis after treatment with active Hla toxin (HlaWt) or HlaPSGS. Graphs report the hemolysis (%) observed following the addition of the three proteins at increasing concentrations. Each point is the mean value of three independent experiments ± SD. (B) Effect of purified anti-HlaPSGS IgG on blood hemolysis induced by wild-type Hla toxin (3.3 μg/ml). Graphs report the hemolysis (%) observed using increasing concentrations of IgG. Each point is the mean value of three independent experiments ± SD. (C) Binding inhibition assay of HlaWt toxin to A549 cells. The binding of 0.3 nM ^35^S-radiolabeled Hla toxin on A549 cells at 5 × 10^5^ cells/500 μl was evaluated after incubation with 15 μl of HlaPSGS antiserum or from sham immunized mice (alum) for 20 min at room temperature. Hla antibody complexes were then incubated for 5 min with A549 cells. Finally, cells were pelleted and added to scintillation fluid for quantification of bound radioactivity (cpm). Significance was determined using Student's *t* test.

Hemolysis is usually considered the hallmark of Hla toxicity and of its pore-forming activity (1). Given that HlaPSGS lacks the stem domain responsible for the formation of the channel across the host membrane, we assessed whether HlaPSGS was impaired in causing hemolysis. Rabbit erythrocytes were mixed with increasing concentrations of either HlaWt or HlaPSGS in a range of 0.001 μg/ml to 50 μg/ml. As expected, HlaWt was able to induce lysis of erythrocytes in a dose-dependent fashion, reaching a plateau of hemolytic activity at a concentration of 0.5 μg/ml (Fig. 5A). However, no lysis was observed in erythrocytes treated with HlaPSGS, even at 50 μg/ml.

Since anti-Hla antibodies neutralize Hla hemolytic activity (26), we then asked whether a similar inhibitory activity was also associated with HlaPSGS antibodies. To this end, erythrocytes were incubated for 20 min with 3.3 μg/ml recombinant HlaWt together with different concentrations of purified rabbit polyclonal IgG against HlaPSGS. A dose-dependent inhibition of hemolytic activity was observed using antibodies against HlaPSGS, reaching 100% inhibition at an antibody concentration of 0.45 mg/ml (Fig. 5B).

Finally, we evaluated whether HlaPSGS-elicited antibodies were also able to inhibit the binding of the wild-type toxin to human lung epithelial cells. The binding of [^35^S]methionine radiolabeled Hla was assessed using A549 cells at 5 × 10^5^ cells/500 μl that were incubated for 20 min at room temperature with 15 μl of either serum collected from HlaPSGS-immunized mice or serum collected from sham-immunized mice (alum). Following a 5-min incubation with radiolabeled Hla, we observed that the presence of serum raised against HlaPSGS (*P* = 0.0016) (Fig. 5C) effectively blocked active toxin binding.

### Antibodies induced by HlaPSGS immunization and S. aureus infection have comparable signatures.

**FIG 6:**
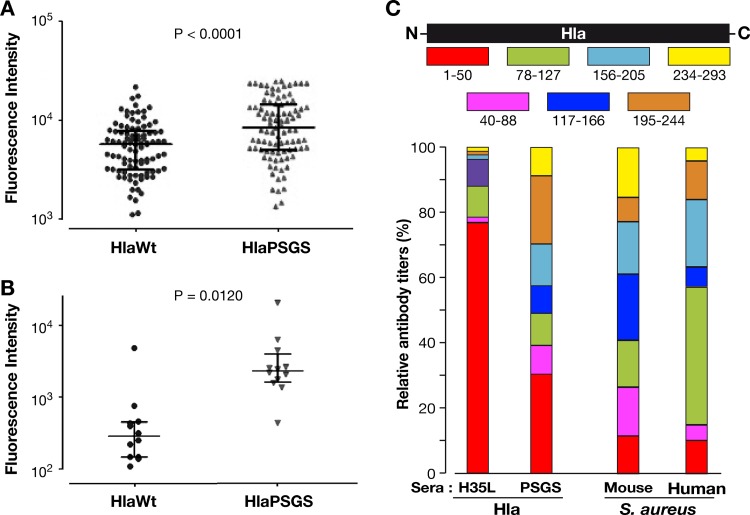
Reactivity profiling of sera from S. aureus-infected mice and human sera toward monomeric and heptameric Hla. Antibody titers against HlaWt and HlaPSGS present in 94 human serum samples (A) and serum samples from 12 S. aureus-infected mice (B). Data points indicate the fluorescence intensity measured by the Luminex assay in each serum, and the bar is the mean value ± standard error of the mean (SEM). Statistical analysis was performed by Mann-Whitney U test. (C) Seven serial 50-amino-acid segments of Hla were purified as GST fusion proteins and were used in the Luminex analysis to map the epitopes recognized by serum samples of different origin (top). Antibody titers against the 7 Hla peptides present in serum samples from 16 mice immunized with HlaPSGS or monomeric Hla (the mutant form HlaH35L, which is unable to form heptamers, was used to immunize mice to avoid toxicity associated with HlaWt) and in 20 human serum samples as well as in serum samples from 12 S. aureus-infected mice. Data are expressed as the median percentage of antibody titers detected against each Hla peptide relative to the total antibody titers measured against the 7 peptides (bottom) (antibody titers are expressed as mean fluorescence intensity [MFI]).

In order to understand whether S. aureus infection elicits antibodies that differentially recognize the monomeric and the heptameric form of Hla, we analyzed 94 serum samples from healthy donors (18 to 81 years old, females and males) for the ability to bind monomeric HlaWt and heptameric HlaPSGS. The analysis was carried out through Luminex technology, immobilizing the two purified proteins on differently labeled microspheres. As shown in Fig. 6A, the serum samples from most of the subjects recognized the heptameric form with higher efficiency than the monomer; the mean fluorescence intensity (MFI) against HlaPSGS (10,375 ± 698) was significantly greater than that of HlaWt (6,192 ± 400). A similar trend was observed when serum samples from mice infected with sublethal doses of S. aureus Newman were analyzed (Fig. 6B).

The above data suggest that upon S. aureus infection, mouse and human immune systems preferentially recognize Hla when it is assembled in the heptameric form on the host cell membrane. This led us to hypothesize that immunization with the monomeric or the heptameric Hla form may induce qualitatively different antibody responses. To test this hypothesis, 7 overlapping 50-amino-acid-long fragments spanning the entire length of Hla (Fig. 6C [9]) were immobilized on microspheres and analyzed by Luminex technology using sera from mice immunized with monomeric Hla and HlaPSGS. Data are expressed as the percentage of antibody titers detected against each Hla peptide relative to the sum of antibody titers measured against the 7 peptides. Therefore, the analysis provides a relative estimate of the titers against each fragment and is independent of the total antibody titers present in the sera. As shown in Fig. 6C, immunization with the monomeric Hla induced antibodies that largely recognized the N-terminal region of Hla. In contrast, anti-HlaPSGS antibodies recognized all seven fragments with similar levels of intensity. Interestingly, the fragment recognition profile of anti-HlaPSGS antibodies resembled the profile observed with sera from S. aureus-infected mice and from human volunteers more closely than that associated with monomeric Hla.

### HlaPSGS vaccination protects mice against S. aureus infection.

**FIG 7:**
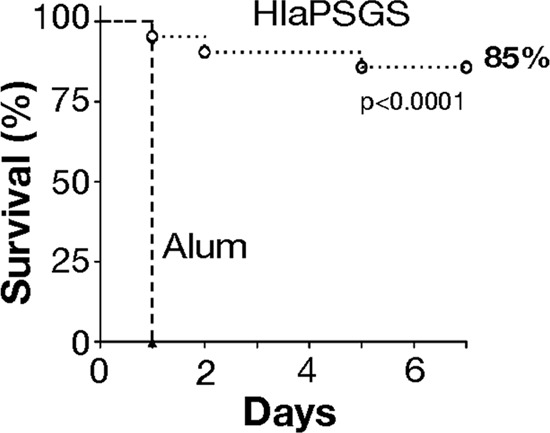
Protective efficacy of HlaPSGS in pneumonia model. Mice were immunized, 2 weeks apart, with HlaPSGS formulated with 2 mg/ml aluminum hydroxide or with the adjuvant alone. Ten days after the second immunization, they were challenged with S. aureus. C57BL/6 mice were challenged intranasally with S. aureus LAC (*n* = 20 per group; 2 separate experiments), and survival was monitored for 1 week. Lines with circles indicate HlaPSGS, and those with triangles indicate mice treated with alum alone. Statistical analysis was performed by log-rank (Mantel-Cox) test.

In order to assess whether HlaPSGS is a suitable vaccine candidate, we investigated whether immunization with this protein provides protection in a mouse pneumonia model of S. aureus infection. HlaPSGS was formulated with aluminum-hydroxide and was used to immunize C57BL/6J mice, while negative controls received identical courses of PBS plus adjuvant (alum). Mice were infected intranasally with S. aureus LAC (USA300) strain, and survival was monitored for 7 days. The pneumonia model and this S. aureus strain were selected because they are considered to be the gold standard for assessing the protective efficacy of Hla antigens (6). Immunization with HlaPSGS exhibited near complete protection, while all sham-immunized animals succumbed on the first day postinfection (Fig. 7).

## DISCUSSION

Hla is a potent toxin produced by most S. aureus isolates and is known to play a key role in S. aureus virulence. Since it has been shown that anti-Hla antibodies neutralize Hla toxicity, it is not surprising that Hla is considered one of the most promising S. aureus vaccine candidates. Over the past few decades, several nontoxigenic mutants have been generated, most of which contain single point mutations that impair heptamer formation, such as the well-characterized HlaH35L mutant (27). More recently, fragments of the protein have been proposed as alternatives to point mutation mutants (7). All of these engineered molecules remain monomeric in aqueous solutions and when exposed to host cell membranes. Therefore, they do not form lytic pores, they are not cytotoxic, and they are considered safe antigens for vaccine use. To our knowledge, heptameric Hla mutants have never before been pursued as vaccine candidates (28).

Since Hla is expected to rapidly form heptameric complexes on host cells when released by S. aureus during infection, this conformation may trigger immune responses different from those elicited by the monomeric mutants used as vaccine candidates. In order to rationally design optimized Hla-based vaccines, it is critical to understand whether there are differences in the anti-Hla antibodies induced by monomeric and heptameric Hla during S. aureus infection.

We generated an Hla mutant that could spontaneously assemble into heptameric complexes in solution. Taking advantage of the availability of the 3D structure of the heptameric Hla wild-type toxin, we created the HlaPSGS mutant, in which the 39-amino-acid stem, spanning from residue Thr^135^ to Val^175^, was removed. In the Hla monomer, this stem shields the surface area involved in monomer-monomer interaction. As predicted, removal of the stem region led to the spontaneous formation of the heptameric complex, even in the absence of cell membrane. This was confirmed by qualitative transmission electron microscopy (TEM) showing that HlaPSGS assembled into a ring-like shaped structure resembling the cap of the Hla wild-type pore. Further single-particle reconstruction analysis applied to the TEM images clearly showed that HlaPSGS is composed of seven identical copies of the same protomer arranged in a C7 symmetry, even in the absence of the stem region.

We then hypothesized that the heptameric conformation of HlaPSGS was able to elicit antibodies with specificity comparable to that elicited by the Hla cytolytic pore. To confirm this hypothesis, antibody titers against wild-type monomeric Hla and HlaPSGS were measured in sera from S. aureus-infected mice. This analysis revealed higher IgG titers against the heptameric form than against the wild-type monomeric protein. A similar pattern was observed with the panel of human sera, suggesting that in humans staphylococcal infection also generates antibodies that better recognize the toxin heptamer than the monomer. Our epitope recognition data showed that antibodies induced by HlaPSGS immunization had an epitope recognition profile similar to that of the sera from donors and S. aureus-infected mice. In particular, the seven Hla fragments were quite uniformly recognized by the sera. In contrast, the sera from animals vaccinated with HlaH35L, which cannot form heptameric complexes, had a more limited recognition profile and a remarkable preference for the N-terminal region of the molecule.

These results demonstrate for the first time to our knowledge that during infection the prevalent form of Hla exposed to the immune system is the heptameric complex. The humoral response against Hla has recently been proposed as a correlate of protection against S. aureus human infections (29, 30). It will be important to understand whether protection is associated with a specific Hla epitope recognition profile of antibodies present in humans and their affinity toward monomeric versus the heptameric form.

At this point, we evaluated the molecule as a vaccine antigen against S. aureus. Interestingly, HlaPSGS was found to be impaired in binding to ADAM10 and human lung epithelial cells. This observation suggests that a preoligomerized toxin is physically unable to bind to the receptor. The second feature represents another significant novelty compared to previously published detoxified mutants that still bind host cells (e.g., HlaH35L [22]). Removal of the stem domain effectively detoxifies Hla and impairs its ability to form the cytolytic pore and to exert hemolytic activity. We then demonstrated that HlaPSGS immunization elicits functional antibodies with neutralizing activity toward Hla-mediated erythrocyte lysis. In addition, HlaPSGS vaccination generated high protective immunity against staphylococcal infection in mice.

In conclusion, the ability of HlaPSGS to self-assemble in liquid solution, its safety profile as a non-pore-forming mutant, and its ability to mimic the immune response generated by infection make this newly engineered Hla molecule an important tool for studying immune responses against the toxin as well as a promising vaccine candidate.

## Supplementary Material

Supplemental material
