# Supplementary material for: Auto-Assembling Detoxified Staphylococcus aureus Alpha-Hemolysin Mimicking the Wild-Type Cytolytic Toxin
Source: Clin Vaccine Immunol. 2016 Jun 6;23(6):442–50. doi: 10.1128/CVI.00091-16 (PMC4895005; doi:10.1128/CVI.00091-16)
Supplement: Supplemental material [file supp_23_6_442__index.html]

Supplemental material 

# Auto-Assembling Detoxified Staphylococcus aureus Alpha-Hemolysin Mimicking the Wild-Type Cytolytic Toxin

## Supplemental material

- Supplemental file 1 -

  Table S1. Primers used in this study.

  PDF, 163K
