# Supplementary material for: Auto-Assembling Detoxified Staphylococcus aureus Alpha-Hemolysin Mimicking the Wild-Type Cytolytic Toxin
Source: Clin Vaccine Immunol. 2016 Jun 6;23(6):442–50. doi: 10.1128/CVI.00091-16 (PMC4895005; doi:10.1128/CVI.00091-16)
Supplement: Supplemental material [file CVI.00091-16_zcd999095352so1.pdf]

Table S1. Primers used in this study

HlaF	CTGTACTTCCAGGGCGCAGATTCTGATATTAATATTAAAACC
HlaR	AATTAAGTCGCGTTAATTTGTCATTTCTTCTTTTTCCCAATCG
HlanatF	GTGCGTCATATGGCAGATTCTGATATTAATATTAAAACC
HlanatR	GCGTCTCGAGTTAATTTGTCATTTCTTCTTTTTCCCAATCG
PSGSF	AATTCGATTGATACACCGTCTGGTTCTGTTCAACCTGATTTCAAAACAA TTTTAGAG
PSGSR	GAAATCAGGTTGAACAGAACCAGACGGTGTATCAATCGAATTTCTTGG ATAGTAATCAG
H35LF	GAAAATGGCATGTTAAAAAAAGTATTTTATAGTTTTATCGATGA
H35LR	ACTTTTTTTAACATGCCATTTTCTTTATCATAAGTGACTAAATC
